# Design and Validation of a Piston-Driven Syringe-Extrusion Bioprinter Using an FDM Frame

**DOI:** 10.3390/biomimetics10120811

**Published:** 2025-12-04

**Authors:** Linlin Zhou, Siheng Su

**Affiliations:** Department of Mechanical Engineering, California State University, Fullerton, Fullerton, CA 92831, USA; linlinzhouyang@outlook.com

**Keywords:** direct ink writing, bioprinting, syringe extrusion, remote piston-driven module

## Abstract

Direct ink writing (DIW) deposits viscous, shear-responsive inks at low temperature, enabling hydrogels and cell-laden bioinks for biomedical fabrication. Access to DIW remains limited by the cost of dedicated systems and the complexity of custom motion control. Repurposing fused deposition modeling (FDM) printers lowers these barriers by using accurate motion stages, open firmware, and familiar workflows while preserving build volume. In this study, three DIW actuator designs were implemented on an FDM frame. The first used a gear-and-rail transmission that converted stepper rotation to plunger travel. The second used a direct trapezoidal-screw pusher that increased force but reduced build-space clearance. The third relocated actuation to a remote piston-driven module that decoupled force generation from the printhead. The final architecture integrates the remote piston with partitioned control, where the printer executes motion and a programmable logic controller (PLC) manages extrusion. This arrangement reduces carried mass, preserves build space, and enables precise volumetric dosing with fast response. On a standard desktop frame, the system achieved controllable deposition of an agar/alginate ink using off-the-shelf electronics and modest modifications. This approach promotes sustainable and accessible innovation by repurposing existing FDM printers with open-source hardware and modular components. The resulting platform supports biomimetic biofabrication by combining mechanical efficiency, environmental responsibility, and cost-effective design.

## 1. Introduction

Additive manufacturing, also called 3D printing, has reshaped product realization by enabling layer-by-layer fabrication with reduced waste, rapid iteration, and digital-to-physical fidelity. This technology was first developed by Chuck Hull in the 1980s, who hardened photopolymers with ultraviolet light to form threedimensional structures, a process now known as stereolithography (SLA) [1]. Today, 3D printing methods include extrusion-based, inkjet-based, and light-based techniques. These approaches span resolutions from the milliscale to the nanoscale, and support a wide range of printable materials and functional geometries that now drive applications in prototyping, tooling, and biomedical devices [2,3,4].

Among these methods, extrusion-based printing is especially attractive for soft matters such as hydrogels. Unlike light-based methods such as SLA or DLP (digital light processing), which rely on low-viscosity photocurable resins and photoinitiators [5], extrusion can process highly viscous, shear-thinning, and cell-laden inks such as agar/alginate blends. This capability allows direct deposition of bioinks with clinically relevant concentrations and cell densities while avoiding light-induced cytotoxicity [6]. Extrusion also enables multi-material and core–shell deposition [7], supporting the fabrication of heterogeneous and vascularized soft tissue constructs. In contrast, SLA and DLP offer superior resolution and rapid printing speeds but are constrained by limited light penetration, scattering within opaque hydrogels, and a narrow selection of compatible photopolymers [8]. Consequently, extrusion bioprinting offers a more versatile and biocompatible platform for fabricating hydrogel-based soft tissues. Hydrogels are widely used for soft tissue fabrication because they closely resemble the native extracellular matrix (ECM) [9]. Their high water content supports the transport of nutrients and oxygen, which promotes cell survival during and after printing. Mechanical properties can be tuned through polymer concentration, crosslinking chemistry, and network architecture so that printed constructs match the softness of many tissues and still retain shape during handling and transplanting [10]. Rheological behavior is critical for printability [11]. Many hydrogel inks exhibit shear-thinning behavior, which reduces extrusion force during flow and then recovers viscosity at rest to preserve strand geometry. Research has demonstrated that 3D-printed hydrogels can satisfy demanding mechanical, biological, and functional targets across many tissues. For example, Namazi et al. [12] reported self-healing and printable hydrogels produced using aldehyde-modified xanthan gum (AXG) and gelatin (Gel) via Schiff base reactions for extrusion-based 3D printing. Three-dimensional structures printed with the optimized formulation (11.3% AXG: 5.7% Gel) exhibited excellent integrity, ~123 μm pores, and thixotropic behavior. It achieved remarkable autonomous self-healing at 37 °C without external stimuli, with an 88% healing efficiency vital for post-extrusion repair. Chu et al. [13] developed a modular bioink for extrusion-based 3D bioprinting, combining gelatin methacryloyl (GelMA) with decellularized extracellular matrix microgels (DMs). The DM-GelMA bioink maintained GelMA’s shear-thinning properties, achieving good printability and structural fidelity. Biologically, DMs offered an ECM-like microenvironment and shear resistance, significantly boosting post-printing cell viability (~86.3%) compared to GelMA alone. García-García et al. [14] developed photocrosslinkable methacrylated chitosan (CHIMe) inks for extrusion-based 3D printing, using N,N′-methylenebisacrylamide (NMBA), polyethylene glycol diacrylate (PEGDA), or acrylic acid (AA) as co-network agents. Simultaneous photocuring with AA resulted in successful printing, overcoming CHIMe’s poor mechanical stability. The optimized inks exhibited excellent printability and offered tunable mechanics, with a Young’s modulus ranging from 14 to 1,068 Pa. These results underscore extrusion’s versatility and biocompatibility for hydrogel-based soft tissue bioprinting.

Extrusion-based bioprinting can be grouped into two primary categories that either process material with melting or dispense material without melting [15]. FDM is a common approach in the first category. During printing, a filament is melted, extruded onto a substrate, and then solidifies into a rigid part. These steps limit the use of FDM for hydrogel printing and other soft tissue constructs because the required temperatures and melt conditions are not compatible with most aqueous bioinks. The second category dispenses material without melting using methods like direct ink writing (DIW) via a syringe-based extrusion technique, which delivers viscous hydrogel inks or precursors at near physiological temperature and can produce cell-compatible constructs [16]. DIW pairs relatively simple, low-cost hardware with the ability to handle higher-viscosity inks than jetting or droplet methods, making it well suited to patient-specific geometries derived from medical imaging. Commercial syringe-extrusion bioprinters like Allevi [17] add sterile enclosures, user-friendly interfaces, and coordinated thermal or light-based crosslinking with customizable process-parameter software. However, these platforms often entail high upfront costs and ongoing software licensing/maintenance fees. Therefore, converting a desktop FDM frame into a syringe-extrusion platform offers a cost-effective alternative to commercial systems and lowers the barrier to entry for exploratory biomedical research and teaching laboratories.

**Figure 1:**
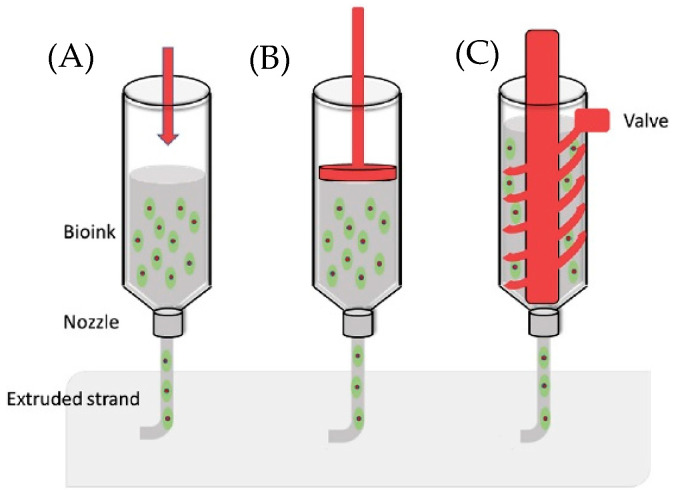
Actuation strategies for syringe extrusion: (**A**) pneumatic; (**B**) piston-driven; (**C**) screw-driven [25].

Actuation strategies for syringe extrusion include two categories: pneumatic and motor-driven (Figure 1) [18]. In pneumatic-driven printers, the pneumatic heads pressurize the syringe plunger with compressed gas and can reach roughly 120 psi in some commercial systems [19]. The high pressure is suitable for tough and viscous inks, but gas compressibility introduces control lag that complicates sharp starts/stops and corner fidelity [20]. Motor-driven systems translate rotary motion from an electric motor into linear plunger displacement. Two common variants are piston-driven and screw-driven. In a screw-driven printhead, the bioink is metered by a rotating screw inside the barrel and is then forced through the nozzle orifice. Screw actuation can provide in situ mixing and can handle higher-viscosity inks, yet the larger pressure drop through the nozzle can stress and damage cells [21]. In a piston-driven printhead, motor rotation is converted to linear travel so the piston advances along the barrel, yielding precise volumetric control with minimal lag and improved feature accuracy [18]. Beyond commercial systems, multiple retrofits of desktop FDM frames demonstrate practical piston-driven DIW: (1) A temperature-controlled, water-jacketed printhead was added to a low-cost 3D printer Anet A8 to tune the rheology of GelMA and Pluronic F-127 for multilayer printing [22]. (2) An open-source, large-volume syringe pump “Bowden-style” extruder accommodates 60 mL syringes, supports retraction to limit oozing, and prints through nozzles as small as 100 µm [23]. (3) A FlashForge Creator Pro was converted to a syringe-based DIW toolhead for soft materials (EcoFlex 00-31 and gelatin–alginate), illustrating straightforward replacement of a thermoplastic extruder with a piston-driven syringe module [24]. These retrofits show that modest mechanical changes and open-source control hardware can reliably convert commodity FDM frames into piston-driven DIW systems. By leveraging off-the-shelf syringes, lead screws, and simple temperature regulation, they reduce cost and complexity while preserving precise flow control, providing a clear, practical pathway for developing and disseminating low-cost DIW bioprinters.

Here, we present a low-cost syringe extrusion bioprinter on a modified FDM frame and demonstrate its printability with hydrogel inks. We designed and tested three interchangeable extrusion heads: (i) a gear and rail transmission that converts stepper rotation to plunger motion, (ii) a direct trapezoidal screw pusher that increases force capacity at the expense of build space clearance, and (iii) a remote piston-driven module that decouples force generation from the printhead to preserve working volume. The final piston-driven configuration provided effective control through coordinated motion and temperature management. A programmable logic controller (PLC) drove the syringe stepper and handled acceleration, deceleration, start, stop, direction change, and homing, while the original printer controller commanded gantry motion. This decoupled architecture allowed volumetric flow to be tuned to ink rheology without changes to the motion firmware. A heated sleeve around the syringe maintained the target printing temperature and offset cooling along the tube, which stabilized viscosity at the nozzle and improved strand diameter uniformity and layer adhesion. Together, the modular heads, decoupled control, and simple thermal regulation provide an accessible path to reliable low-cost DIW bioprinters.

The printability was assessed with agar/alginate blends as a representative double network precursor. Agar and alginate are naturally derived polysaccharides that are widely employed in biomedical engineering for their biocompatibility, biodegradability, and non-immunogenicity [26]. They are widely reported as cytocompatible materials for extrusion-based printing and cell encapsulation [27,28], which motivated their use as a test ink in this study. Inks were prepared at defined concentrations and dispensed through the extended needle. Layer height was selected relative to the needle’s inner diameter to maintain strand continuity and to control overlap between adjacent rasters. It is shown that the system reproduced the intended shapes with good fidelity, which supports the suitability of the platform for soft tissue constructs and for future studies that include cells or bioactive cues. Beyond demonstrating printability and control performance, this study also advances sustainable and accessible innovation in biomimetic fabrication. By repurposing an existing FDM printer frame with open-source hardware and modular components, this approach reflects the biomimetic principle of efficiency through adaptation, utilizing available resources to achieve functional complexity with minimal inputs. The resulting system offers a reproducible, environmentally responsible, and economically accessible route to hydrogel-based biofabrication that bridges mechanical design, materials engineering, and biomimetic research.

## 2. Materials and Methods

### 2.1. Preparation of Bioink

Agar powder (C_12_H_18_O_9_)n and sodium alginate were purchased from MilliporeSigma (Burlington, MA) and used as received. Sodium alginate (250 mg) was dissolved in 50 mL deionized water under gentle stirring overnight at room temperature. In parallel, agar (250 mg) was dispersed in 50 mL deionized water and heated to 90 °C with continuous stirring overnight until the solution became fully transparent with no suspended particles. The alginate solution was then added dropwise into the hot agar solution while maintaining 90 °C, and the mixture was stirred until homogeneous. The resulting composite is hereafter referred to as the agar/alginate blend. The composite ink was used immediately for printing trials or transferred to a preheated syringe for subsequent use.

### 2.2. Viscosity Measurement

Apparent viscosity was measured with a Brookfield DVE rotational viscometer. Agar, alginate, and mixed agar/alginate inks were equilibrated at the targeted temperature, loaded into the sample cup, and measured after the reading stabilized. Shear rate was swept from 0 to 140 rpm at room temperature. Temperature sweeps were conducted from 40 °C to 100 °C at 100 rpm, with samples re-equilibrated at each set point before measurement.

### 2.3. Injection Load Test

A benchtop Mark-10 tensile/compression tester was used in compression mode to estimate the plunger force required for syringe extrusion prior to finalizing the drive design. A luer-lock syringe was filled with 20 mL of the agar/alginate blends, de-bubbled, capped with the same needle/tubing set planned for printing, and mounted in a rigid axial fixture. The tester’s compression platen was aligned to the syringe plunger with a flat pusher to avoid side loads. The crosshead speed was set to 1 mm/min. Injection force vs. time data were recorded continuously.

### 2.4. Plunger Force Estimation

Analytical predictions of available plunger force were computed from motor torque and transmission geometry. Motor torque at the intended extrusion speed was taken from the torque versus speed curve or measured at constant speed. Transmission efficiency was estimated from published ranges.

Design 1 used a rack and pinion on the carriage. The predicted plunger force was

$$F=\eta \frac{T_{motor}}{r_{pinion}}$$

where $T_{motor}$ is the motor torque at speed in N·mm, $r_{pinion}$ is the pinion pitch radius in mm, and η is the mechanical efficiency. The torque of the motor used in Design 1 is 420 N·mm, the pinion radius is 15 mm, and the mechanical efficiency is assumed to be 88~98% [29].

Design 2 and Design 3 used trapezoidal lead screws. The predicted plunger force was

$$F=\eta \frac{2\pi T_{motor}}{lead}$$

where $T_{motor}$ is the motor torque at speed in N·mm, lead is the screw advanced per revolution in mm/rev, and η is the overall efficiency. The torque of the motor used in Design 2 is 420 N·mm, the lead size is 8.0 mm/rev, and mechanical efficiency is assumed to be 10.9~33.6% [30]. In Design #3, the torque of the motor is 1100 N·mm and the other parameters are the same as those of Design #2.

### 2.5. Control Partition and Electronics

In Design #3, extrusion actuation was separated from gantry motion using a decoupled control architecture. The original printer controller continued to command XYZ motion, while a PLC drove the syringe stepper motor. The PLC program implemented operator controls for acceleration, deceleration, start, stop, homing, and direction switching through dedicated inputs, allowing the extrusion rate to be tuned to ink rheology without altering motion firmware. The PLC and drive electronics were integrated with the remote piston-driven module so that force generation was offloaded from the printhead, preserving working volume on the modified desktop FDM frame.

### 2.6. Prototype Printing

Structural and interface components for the retrofit, including the syringe carriage, gear and rail body, trapezoidal screw pusher mount, remote piston housing, nozzle holder, and frame adapters, were fabricated on an Ultimaker 2 using 2.85 mm PLA filament. STLs were sliced in Cura with a 0.4 mm nozzle, 0.2 mm layer height, 100% infill, nozzle temperature 200~210 °C, bed temperature 60 °C, cooling after layer 2, and supports/brim as needed.

## 3. Results

In this paper, the platform evolved through three designs that progressively increased force margin and reduced compliance. The modified FDM frame was an Ultimaker Original+ 3D printer (Ultimaker, B.V., Utrecht, The Netherlands). Design 1 placed a gear and rail transmission on the carriage. Transmission stiffness was limited and backlash was evident. Continuous strands were possible only at a low mixture concentration, which did not retain its shape after deposition. Design 2 replaced the gear and rail with a trapezoidal screw that acted directly on the plunger. The available thrust increased and higher-viscosity inks could flow. The longer assembly intruded into the build volume and increased moving mass. Design 3 relocated actuation off the carriage to a remote piston-driven module. Working volume was restored, moving mass at the printhead decreased, and compliance in the force path was reduced. Retraction became feasible.

### 3.1. Design 1: Gear and Rail Transmission

**Figure 2:**
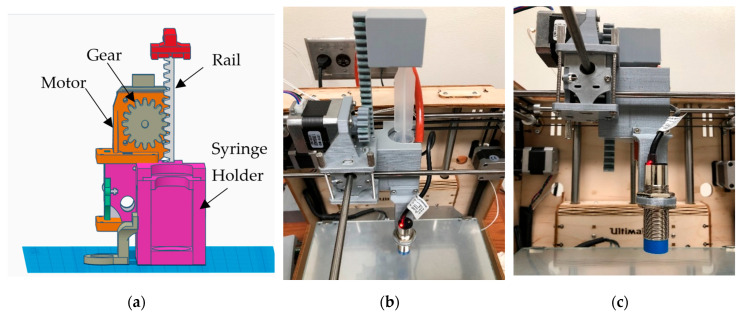
Syringe toolhead for Design 1: (**a**) CAD rendering; (**b**,**c**) photographs. (Reproduced from [31].)

The first prototype converted stepper motor rotation into linear plunger motion using a printed gear-and-rail (rack-and-pinion) mechanism, and all custom parts were fabricated in PLA on an Ultimaker 2+ (Ultimaker, B.V., Utrecht, The Netherlands). First, the thermoplastic hot end of the Ultimaker Original+ was removed and replaced with a syringe-based extrusion head (Figure 2). Specifically, a printed PLA clamp with locking lips secured the syringe barrel, one with a simple heated sleeve stabilized barrel temperature, and a long needle served as the dispensing nozzle. The assembly mounted to the Ultimaker XY carriage using the original linear bearings. A small bracket co-located the capacitive probe with the needle tip to support automated Z-offset calibration. A carriage-mounted NEMA-17 motor (Wantai Motor, Shanghai, China) drove a spur gear that engaged a printed rack. The rack pushed a latched pusher linked to the syringe plunger, producing the required linear extrusion stroke. Firmware coordinate offsets and soft travel limits were updated for the new toolhead geometry, and motion profiles were tuned for slow, uniform feed to accommodate semisolid flow.

**Figure 3:**
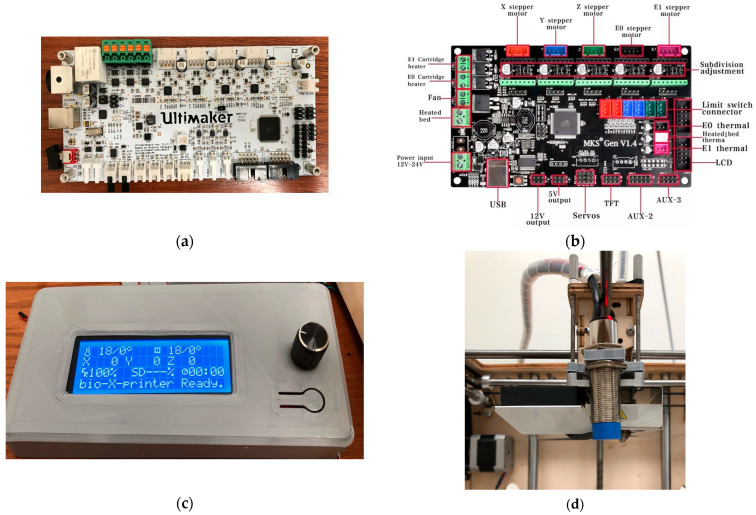
Control hardware and sensing: (**a**) stock Ultimaker motherboard; (**b**) MKS GEN V1.4 [32]; (**c**) RepRap Discount Smart Controller; (**d**) capacitive proximity sensor near the needle.

To enable flexible motion control and standalone operation, the stock controller was replaced with an MKS GEN V1.4 running Marlin (Figure 3). The upgrade enabled firmware-level control of motion limits, PID temperature regulation, bed probing, and connected peripherals. It also supported SPI-addressable stepper drivers such as TMC2130. To improve calibration, a capacitive proximity sensor was added for automatic bed leveling. For more stable temperature feedback, the stock Pt100 sensors were replaced with 100 kΩ thermistors. Finally, a RepRap Discount Smart Controller was integrated to allow untethered SD card printing.

With the MKS GEN V1.4/Marlin stack, the system gained (i) firmware-set motion bounds and PID temperature control; (ii) SPI stepper driver configuration; (iii) auto bed leveling via capacitive proximity sensing; and (iv) 100 kΩ thermistor inputs in place of Pt100. The RepRap Discount Smart Controller (Makerbase Technology Co., Ltd., Shenzhen, China) (20 × 4 LCD with a rotary encoder and onboard SD) provided fully untethered operation displaying printer/SD status, hot end and bed temperatures, print timer, speed, and XYZ coordinates, as well as error messages (e.g., thermal faults or driver errors). A hardware reset button allowed quick recovery from faults. With the panel integrated, calibration, axis jogging, and preheat could be performed without a host PC, and prints ran directly from G-code on the SD card.

The design was capable of extruding low-apparent-viscosity agar/alginate blends that fell below the printable viscosity range for extrusion-based bioprinting (approximately $30\sim 6\times 10^{7}\;\mathrm{mPa}\cdot s$ [33]. Also, the printed transmission exhibited insufficient stiffness and noticeable backlash, limiting peak extrusion force and dosing precision. Practically, agar/alginate blends above ~3 g/L could not be driven reliably. At these lower concentrations, the strands lacked shape fidelity after deposition [34], and force losses through the compliant, back driving rack-and-pinion produced under-extrusion and poor print quality.

### 3.2. Design 2: Direct Screw Pusher (Elongated Variant)

**Figure 4:**
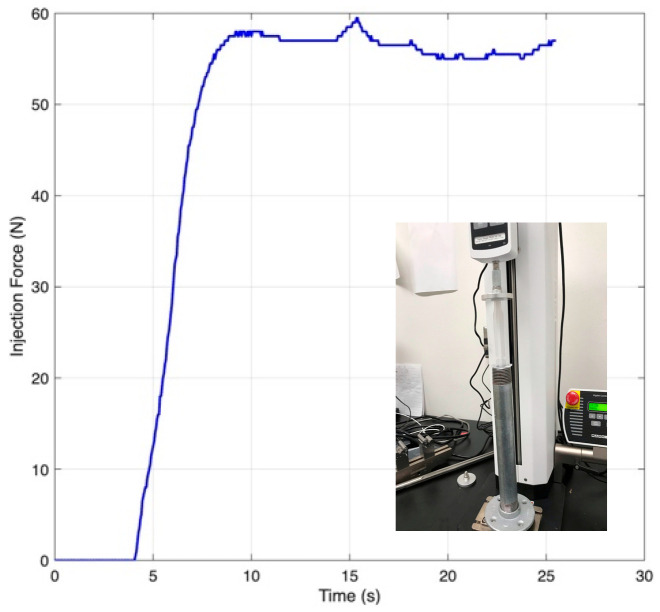
Plunger force vs. time during injection of an agar/alginate blend (adapted from [31]).

A compression test using a 20 mL syringe filled with an agar/alginate blend established the minimum drive force needed for steady flow through the dispensing needle. The measured injection trace (Figure 4) showed a lower bound thrust of ≈58 N, which was adopted as the sizing target for subsequent actuators.

**Figure 5:**
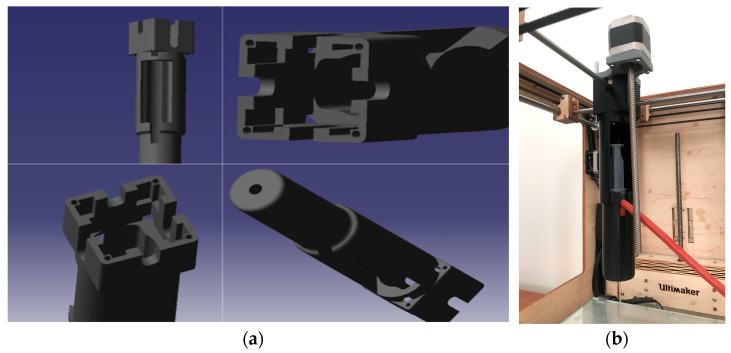
Direct screw pusher (Design 2): (**a**) CAD rendering; (**b**) assembled toolhead showing the elongated screw/plunger stack. (Reproduced from [31].)

To meet the load with less compliance than the rack and pinion in Design 1, the second prototype drove the syringe plunger directly with a stepper-driven trapezoidal lead screw, in which a traveling nut bore against a plunger yoke to generate linear push (Figure 5). As with the first prototype, the stock Ultimaker controller was replaced by an MKS GEN V1.4 running Marlin (Makerbase Technology Co., Ltd., Shenzhen, China) to simplify firmware changes (e.g., offsets and soft limits) during integration and tests.

The direct screw pusher delivered the desired high thrust, comfortably exceeding the 58 N force requirement and enabling extrusion of higher-viscosity bioinks by eliminating transmission lash and flex. However, the stacked length of the syringe barrel, plunger, screw, and nut created an unusually long, front-to-back toolhead. In practice, the assembly intruded into the gantry’s travel envelope, reducing usable build volume and interfering with head motion. Thus, the limitation was packaging, not force.

Given these interferences, the next iteration decoupled the heavy extrusion hardware from the printhead, which could retain high thrust while restoring working volume by delivering material through tubing to a compact nozzle.

### 3.3. Design 3: Remote Piston-Driven Module

**Figure 6:**
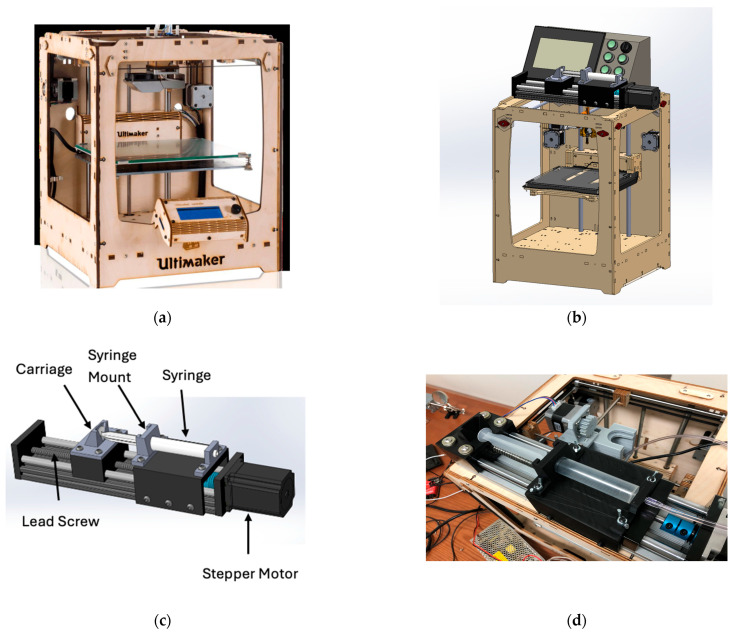
(**a**) Remote piston-driven module on a modified FDM frame (Design 3): (**a**) Ultimaker Original+ [35]; (**b**) full bioprinter CAD; (**c**) extrusion module CAD; (**d**) assembled extrusion module. (Reproduced from [31].)

In the third design, to recover working volume and improve dynamic response, the syringe actuator was moved off the carriage and built as a self-contained “remote piston” module (Figure 6). A stepper-driven trapezoidal lead screw on a guided squeeze track advanced a pusher that engaged the plunger, while a two-piece syringe holder rode on smooth rails: the barrel half fixed to the rail, and the pusher half coupled to the screw’s traveling nut. Material exited the stationary syringe through a long tube to a small nozzle on the printhead, so the XY gantry carried only the lightweight needle and probe. Relocating mass off the carriage restored usable build space and, by shortening the compliant elements in the force path, improved start/stop behavior and enabled reliable retraction via commanded direction reversals.

**Figure 7:**
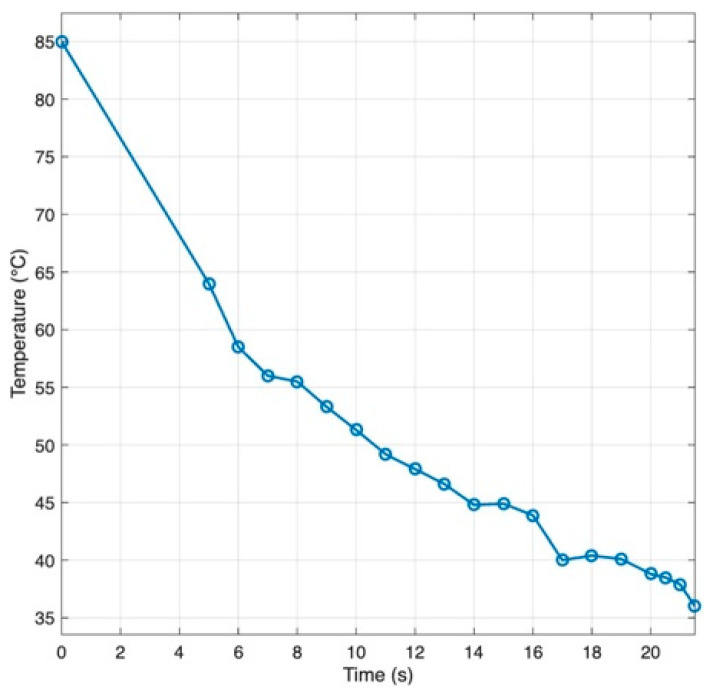
Temperature drop along the syringe to nozzle path for a agar/alginate blends (Adapted from [31].)

Because material traveled from the heated syringe through tubing to the nozzle, temperature transients were characterized to define a timing “lead”. As shown on Figure 7, with a heated sleeve on the syringe, the representative agar/alginate blend cooled from ~85 °C at the barrel to the 60 °C printing set point in about 6 s by the time it reached the nozzle. Operationally, extrusion was triggered ~6 s before toolpath arrival so that flow onset coincided with the nozzle reaching the start point.

**Figure 8:**
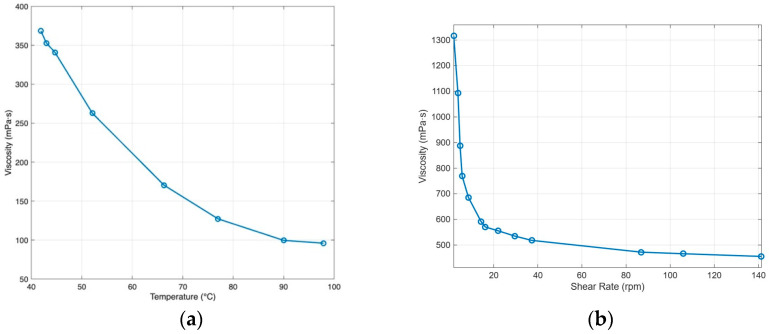
Rheology of agar/alginate: (**a**) apparent viscosity vs. temperature at 100 rpm; (**b**) apparent viscosity vs. shear rate at room temperature. (Adapted from [31].)

The rheology of the 5 g/L agar/alginate ink depends on both the shear rate and the temperature. Figure 8 demonstrated that the ink shows clear shear thinning at room temperature, with viscosity dropping quickly as the shear rate increases. This allows easy flow through the nozzle and recovery of viscosity once the shear stops. Viscosity also falls as temperature rises. For the working blend, the apparent viscosity is about 220 mPa·s under 60 °C at a shear rate of 100 rpm and it continues to decline with further heating, while cooling produces a sharp increase. During printing, the syringe and nozzle should be kept warm enough to maintain consistent flow, but not so warm that deposited strands lose definition. After deposition, controlled cooling keeps each filament warm long enough to fuse with the previous layer, then allows gelation to hold the shape. This balance prevents clogging and under-extrusion and preserves dimensional accuracy.

**Figure 9:**
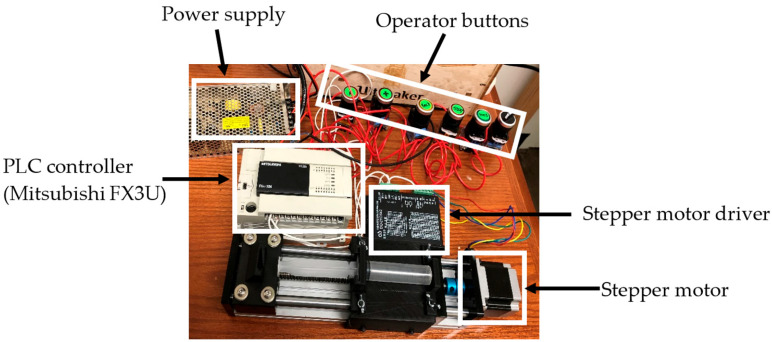
PLC-driven extrusion subsystem: photos of the Mitsubishi FX3U PLC, stepper driver and motor, operator buttons, and power supply (adapted from [31]).

Extrusion was decoupled from the printer’s motion firmware and driven by a PLC that commanded the syringe stepper independently of the gantry (Figure 9). Front-panel controls provided acceleration, deceleration, start/stop, quick homing, and direction switching (used for clean retractions). This separation allowed the extrusion rate to be tuned to the ink rheology without modifying motion firmware. A high commanded extrusion speed without sufficient thermal conditioning produced a characteristic failure mode documented during testing, reinforcing the need to operate within the rheology window defined above and to respect the temperature lead time observed along the feed path.

**Figure 10:**
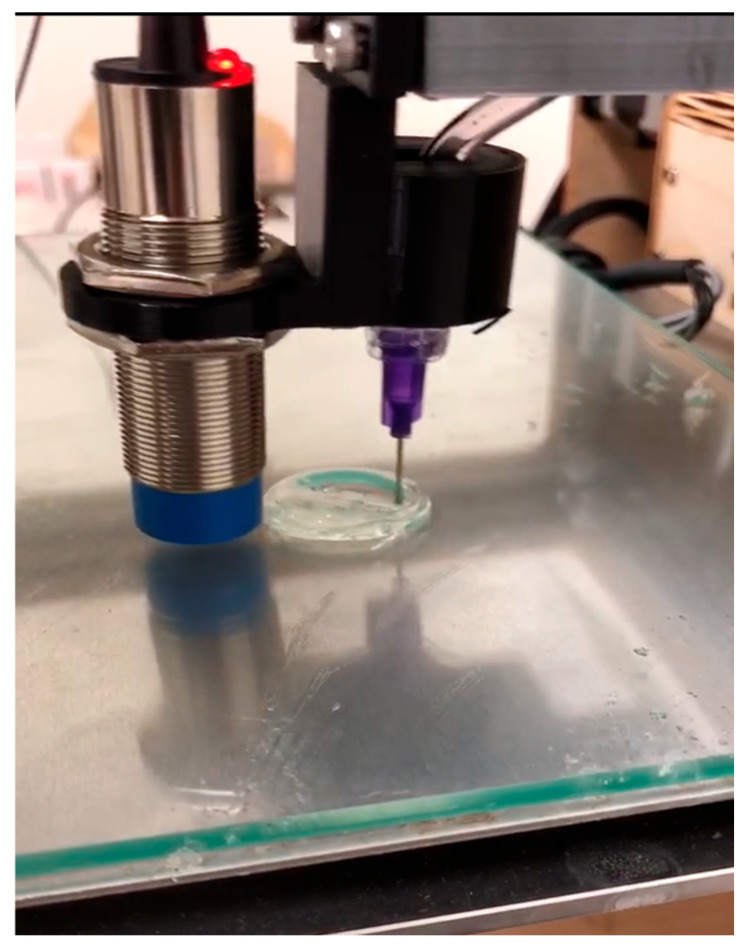
Printability study of agar/alginate ink with 21-gauge nozzle at 0.6 mm layer height showing single-filament and perimeter paths. The image captures partial layers during deposition (reproduced from [31]).

Deposition trials were performed with agar/alginate blends using the remote piston configuration. A 21-gauge needle was used, and slicing employed a 0.6 mm layer height to match the dispensing scale (Figure 10, Video S1). Trials consisted of single filaments, short perimeters, and partial layers. Continuous strands were produced with stable start and stop when the extrusion trigger led the toolpath by ~6 s. This lead was empirically calibrated for the tested setup to offset cooling along the syringe-to-nozzle path. Because the transfer line is unheated, retraction is not universally guaranteed. In our tests with a 5 g/L agar/alginate blend operated near 60 °C, short retractions were feasible and improved starts/stops. However, at lower temperatures or with higher-viscosity inks the risk of cooling-induced loss of flow increases. The platform therefore demonstrated controllable strand placement within the identified thermal and rheological window, while further tuning of the temperature conditioning and feed rate is needed to complete enclosed features, and a heated transfer line is a logical future enhancement.

### 3.4. Comparative Summary of the Three Designs

Across the iteration sequence, measured and observed performance improved monotonically in thrust margin, dynamic response, and usable build space (Table 1). Design 1 was limited by transmission stiffness and could not meet the force target for higher viscosity inks. Design 2 met thrust requirements but compromised working volume due to its overall length. Design 3 delivered the required thrust while preserving build space and reducing compliance, enabling controllable strand placement under the tested conditions.

## 4. Discussion and Conclusions

Relocating syringe actuation into a remote piston-driven module and partitioning control between the printer for motion and the PLC for extrusion addressed the principal limitations encountered with carriage-mounted designs, namely excess moving mass, reduced build volume, and unreliable starts and stops. In deposition trials with a 21-gauge needle and 0.6 mm layer height, continuous strands with stable starts/stops were obtained when the extrusion trigger preceded toolpath arrival by ~6 s to offset cooling and transport lag from syringe to nozzle. In this work, the ~6 s value is an empirical calibration outcome for the tested configuration (5 g/L agar/alginate, heated syringe, unheated transfer line) rather than a universal constant. Because the transfer line is unheated, retraction is not universally guaranteed: in our tests near 60 °C, short retractions were feasible and improved starts/stops, whereas longer pauses or large reverse strokes increased the risk of cooling-induced loss of flow. Accordingly, we limited retraction distance and dwell, used coasting/wipe moves, and minimized idle time. Within this window, the platform demonstrated controllable strand placement.

This work demonstrates a practical and reproducible route for DIW bioprinting on desktop hardware. Several limitations remain. Temperature regulation is open-loop at the syringe, pressure in the feed path is not measured, and coordination between PLC-driven extrusion and gantry motion is based on a simple trigger. Importantly, the timing lead is a calibrated value that varies with tubing length/ID, flow rate, nozzle gauge, ambient temperature, and ink rheology. A practical procedure is to equilibrate the syringe, extrude into air to measure time-to-steady flow at the nozzle, use the mean as the initial lead, and adjust in small increments during the first path to synchronize flow onset with toolpath arrival. Future work will add a thermocouple near the nozzle and a closed loop controller to stabilize strand diameter during long moves. An inline miniature pressure transducer will be integrated to calibrate volumetric flow and to tune retraction in order to limit oozing. Extrusion setpoints will be synchronized with motion through a digital signal so that starts and stops align with toolpath position and cornering, which should reduce over-extrusion at corners and improve fidelity. Thermal management along the feed path will be strengthened, and the process window for cell-laden formulations will be mapped to link printing parameters with biological outcomes. A sterile enclosure and a multi-material manifold are also planned to broaden relevance to work with living cells while preserving the low cost and reproducible path of the present design.

## Figures and Tables

**Table 1:** Comparison of major attributes for the three design configurations.

Attribute	Design 1	Design 2	Design 3
Transmission	Gear and rail on carriage	Trapezoidal screw on carriage	Remote trapezoidal screw on guide rails
Estimated plunger force (N)	27-9	36-111	94-290
Added mass on carriage	Moderate	Larger than design one	Low
Supported syringe volume * (mL)	5 mL	10 mL	20 mL
Retraction feasibility	No	Limited	Yes

* Supported syringe volume. Maximum nominal syringe size that fits the holder and tubing.

## Data Availability

The raw data supporting the conclusions of this article will be made available by the authors on request.

## Supplementary Materials

The following supporting information can be downloaded at https://www.mdpi.com/article/10.3390/biomimetics10120811/s1, Video S1: Printability study of agar/alginate ink at 0.6 mm layer height with a 21-gauge needle.

## Abbreviations

The following abbreviations are used in this manuscript:

AA	Acrylic acid
AXG	Aldehyde-modified xanthan gum
CAD	Computer-aided design
CHIMe	Methacrylated chitosan
DIW	Direct ink writing
DLP	Digital light processing
DM	Decellularized matrix
ECM	Extracellular matrix
FDM	Fused deposition modeling
GelMA	Gelatin methacryloyl
I/O	Input/output
LCD	Liquid crystal display
PEGDA	Polyethylene glycol diacrylate
PID	Proportional integral derivative
PLA	Polylactic acid
PLC	Programmable logic controller
SLA	Stereolithography
SPI	Serial peripheral interface
